# Anesthetic gases environmental impact, anesthesiologists’ awareness, and improvement opportunities: a monocentric observational study

**DOI:** 10.1186/s44158-024-00183-1

**Published:** 2024-07-25

**Authors:** Ludovico Furlan, Pietro Di Francesco, Patrick Del Marco, Jacopo Fumagalli, Chiara Abbruzzese, Giacomo Grasselli

**Affiliations:** 1 Department of Internal Medicine, Fondazione IRCCS Ca’ Granda Ospedale Maggiore Policlinico, Milan, Italy; 2 Department of Clinical Sciences and Community Health, University of Milan, 20124 Milan, Italy; 3 Department of Primary Care, ASST Valtellina, Sondrio, Italy; 4 Department of Anesthesia, Intensive Care and Emergency, Fondazione IRCCS Ca’ Granda Ospedale Maggiore Policlinico, Milan, Italy; 5 Department of Pathophysiology and Transplantation, University of Milan, Milan, Italy

To the Editor,

Healthcare systems contribute to 5–10% of national greenhouse-gas emissions [1], with anesthetic gases (AG) accounting for 2–5% of such emissions [2]. Position papers recommend considering, whenever feasible, total intravenous anesthesia (TIVA) over inhalational anesthesia since it is almost 10^4^ times less polluting [3–6].

We investigated, at our university center, if AG use has decreased over the years, how frequently anesthesiologists consider TIVA an appropriate alternative to AG, and what is their awareness on the environmental impact of AG.

First, using the electronic records of the operating rooms’ pharmaceutical orders, we extracted the amount of general anesthetics used between 2017 and 2022 and verified if AG use changed over time. To account for monthly variations in orders, we compared aggregate data per semester. To account for reduction of surgical procedures during COVID pandemic, we normalized AG use per hour of intervention performed. We did not review the single procedures but simply divided total amount of medications ordered per total hours of interventions performed. We also compared, using previously published data [4, 7], the environmental impact attributable to AG and propofol for 2022.

In the second part of the study, we invited anesthesiologists of our institution to fill an anonymized survey on Google Form (Google, California, USA) and recorded data on their experience, surgical procedures assisted, and habits regarding AG and TIVA. As primary outcome, we asked anesthesiologists how often, based on their clinical experience, TIVA might substitute AG, choices being < 20%, 20–40%, 40–60%, and > 60%. We then provided data on the environmental impact of AG and asked to express awareness on the issue on a scale from 1 to 6 (1 = not aware, 6 = totally aware).

We analyzed 47,908 surgical procedures and 1343 pharmaceutical orders. Use of AG did not vary over the years and caused 99.93% of the 178 tons of CO2_e_ attributable to general anesthetics in 2022. Desflurane caused 42.2% of emissions despite being used 10 times less than sevoflurane. To compensate such emissions, almost 3000 tree seedlings grown for 10 years would be needed (Fig. 1).

**Fig. 1:**
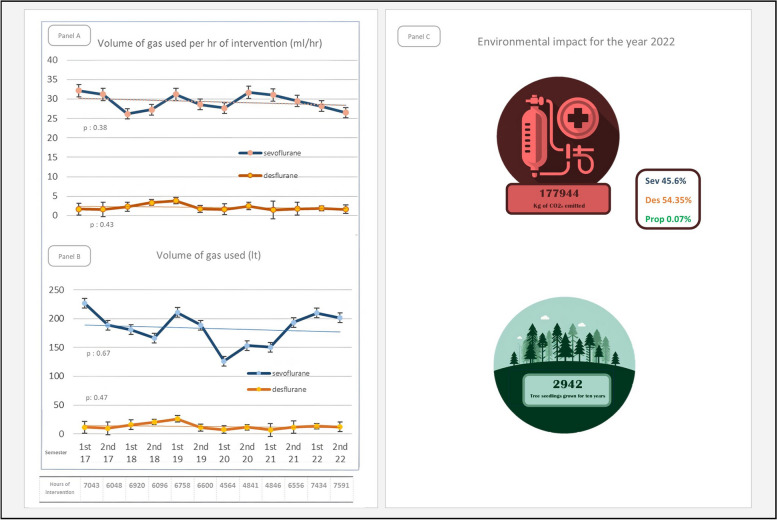
Variation of AG use from 2017 to 2022 and environmental impact from general anesthetics for the year 2022. **A** and **B** Report variation of general anesthetics use throughout semesters from 2017 to 2022 considering respectively volume of AG used per hour of intervention performed and total volume of AG used. The variation of use of AG was analyzed through a linear regression model. For each measure, *p*-value for statistical significance considering the slope coefficient of the linear model is reported. **C** Reports the emissions attributable to general anesthetics for the year 2022 and contribution from each medication. At our center, nitrous oxide use is negligible, while isoflurane is not available. The environmental impact of AG and propofol for the year 2022 is expressed as equivalent tons of CO2 (CO2e) and was calculated using previously published data [4]. The number of trees needed to compensate such emissions was calculated using the US Environmental Protection Agency calculator [7]. Sev, sevoflurane; Des, desflurane; Prop, propofol

For 85.4% of interviewed anesthesiologists, the most used drug was sevoflurane, followed by propofol for 12.2% and desflurane for 2.4%. Most anesthesiologists declared that TIVA could substitute AG more than 40% of times, while only 5% thought this could happen in less than 20% of cases. Sixty-five percent of anesthesiologists were partly or totally unaware of AG environmental impact (Table 1).

**Table 1:**
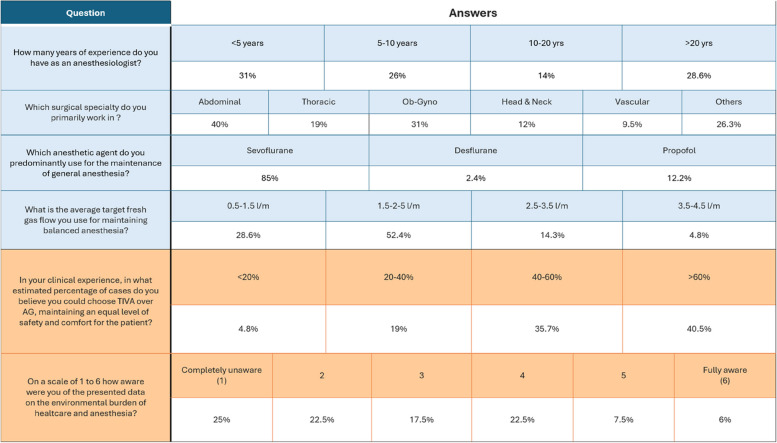
Survey respondents' characteristics (blue) and answers to the main outcomes of interest (orange) Percentages refer to the rate of interviewees that provided the answer

Despite several calls for action, at our center, AG use has not changed in recent years, causing large amounts of emissions. The awareness of the environmental impact of AG is limited, but, according to anesthesiologists’ judgement, there is room for shifting from AG to significantly less polluting medications in more than 40% of cases. Position papers recommend abandoning desflurane and limiting fresh gas flow for maintenance of anesthesia as effective mitigation strategies [3, 6]. Our data confirm that avoiding desflurane use could have a great environmental impact, and that frequently more than 1.5 l/min of fresh gas flow is used to maintain anesthesia.

## Data Availability

Administrative data on medication used and total hours of intervention or property of La Fondazione IRCCS Cà Granda Ospedale Maggiore Policlinico, Milan, Italy. Data access is subject to prior approval and authorization from La Fondazione IRCCS Cà Granda Ospedale Maggiore Policlinico. Original data from the anesthesiologists’ questionnaire may be provided, on request, by the corresponding author.
